# Comparison of Postoperative Outcomes Following Medial Parapatellar Versus Medial Subvastus Approaches for Total Knee Arthroplasty in a Single Patient: A Case Report

**DOI:** 10.7759/cureus.88843

**Published:** 2025-07-27

**Authors:** Jason S DeFrancisis, Rachel Munro, Paul Danahy, Mark E Farmer

**Affiliations:** 1 Orthopedic Surgery, Lake Erie College of Osteopathic Medicine, Bradenton, USA; 2 Orthopedic Surgery, Orthopedic Center of Florida, Fort Myers, USA

**Keywords:** knee osteoarthritis (koa), medial parapatellar approach, orthopaedic surgery, orthopedic surgery, patient recovery, postoperative outcomes, subvastus approach, tka, total knee arthroplasty (tka)

## Abstract

Osteoarthritis (OA) is a chronic degenerative condition that impacts the joints, most commonly the knee. The definitive treatment for severe knee OA is surgery with a total knee arthroplasty (TKA), which is traditionally done through a medial parapatellar approach; however, alternative approaches, including the medial subvastus approach, can be utilized. This case compares the postoperative outcomes between the medial parapatellar and medial subvastus TKA approaches. A 68-year-old male with a past surgical history of a right knee TKA presented to the orthopedic clinic with a several-month history of left knee pain, which worsened with walking and prolonged standing. X-ray revealed severe left knee OA, and the patient opted to undergo surgical fixation as prior conservative management had failed. However, prior to surgery, the patient expressed concern as he had a difficult postoperative course following a previous right knee TKA that was done with a medial parapatellar approach. In an attempt to improve early postoperative recovery, the patient underwent a left knee TKA with a medial subvastus approach. The patient had an uncomplicated procedure followed by a remarkable postoperative recovery that consisted of minimal pain, early ambulation, and early use of stairs. This case underscores the value and potential advantages of the medial subvastus approach in TKA, particularly for patients with a history of challenging postoperative recovery following a traditional medial parapatellar approach.

## Introduction

Osteoarthritis (OA) is a long-term, degenerative condition that impacts the joints [1]. The knee is the most commonly affected joint from OA, with an incidence of 203 per 10,000 individuals [2]. Knee OA can be classified as either primary or secondary, depending on its etiology [1]. Primary knee OA typically occurs without a clear underlying reason and is often linked to aging or a genetic predisposition [1]. In contrast, secondary knee OA develops as a result of identifiable factors such as joint injury, congenital abnormalities, or other underlying health conditions [1]. The majority of cases of knee OA are primary OA and occur in patients with risk factors such as old age, high BMI, female sex, and prior knee injury [1,3].

Knee OA typically presents as knee pain that worsens with activity and improves with rest [4]. Stiffness, reduced range of motion, joint deformity, and weakness often lead to ambulatory disabilities, and all are common presenting features as well [4]. Knee OA is diagnosed based on clinical presentation, physical examination, and imaging [5]. X-ray is the first-line imaging modality through bilateral weight-bearing anteroposterior (AP), lateral, and axial patellar views [1]. Common radiologic features of knee OA include joint space narrowing, subchondral sclerosis, osteophytes, and subchondral cysts [6]. The severity of knee OA is assessed using the Kellgren-Lawrence classification, a 0-4 grading system based on weight-bearing AP X-rays [6]. Treatment approaches to knee OA vary, ranging from conservative to surgical intervention, with surgical intervention being the definitive treatment [7,8]. Conservative management of knee OA includes weight loss, physical therapy, pharmacological intervention such as topical and oral non-steroidal anti-inflammatory drugs, as well as intra-articular corticosteroid, hyaluronic acid, and platelet-rich plasma injections [7]. As for surgical intervention, total knee arthroplasty (TKA) is the standard definitive treatment and is utilized for advanced knee OA [8]. Complications of untreated knee OA consist of progressive radiographic changes, worsening pain, poor joint stability, and significant functional decline, with most patients eventually unable to walk [9].

The traditional TKA approach is the medial parapatellar approach; however, alternative approaches are occasionally utilized in practice [10]. The medial subvastus approach is an alternative approach to the TKA that can have benefits in the early postoperative recovery course [11]. This case highlights a patient who underwent a TKA via the medial parapatellar approach on one knee, followed by a contralateral TKA using a medial subvastus approach. This case report aims to compare postoperative outcomes between the two techniques and inform orthopedic surgeons of the potential advantages of the medial subvastus approach, particularly for patients with difficult recoveries after prior TKA.

## Case presentation

**Figure 1:**
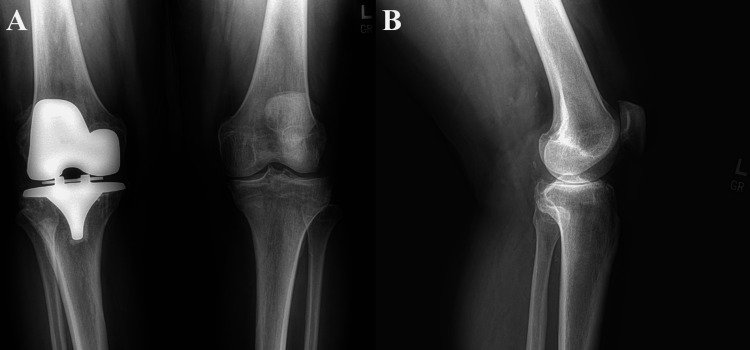
(A) Preoperative anteroposterior radiograph of the knees demonstrating right knee total arthroplasty and left knee osteoarthritis. (B) Preoperative lateral radiograph of the left knee demonstrating osteoarthritis. (A) Preoperative anteroposterior X-ray of both knees demonstrates a well-positioned total knee arthroplasty in the right knee with stable osseous integration and no signs of loosening. The left varus knee demonstrates severe medial compartment osteoarthritis with joint space narrowing, subchondral sclerosis, and osteophyte formation, with no acute fractures identified. (B) Preoperative lateral X-ray of the left knee demonstrates osteoarthritis with joint space narrowing, subchondral sclerosis, and osteophyte formation, with no acute fractures identified.

**Figure 2:**
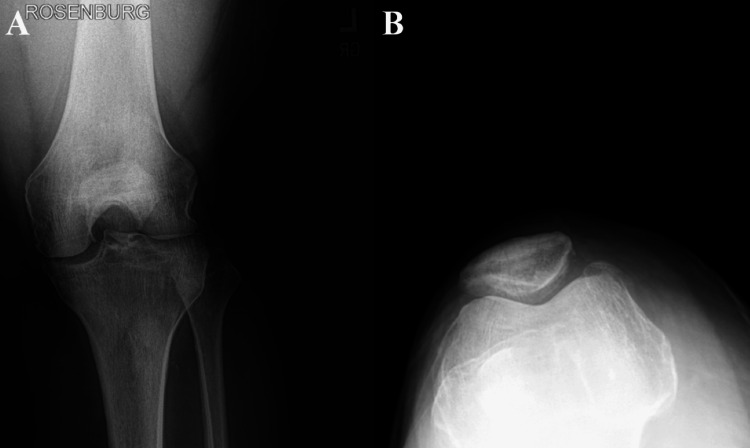
(A) Preoperative axial view of the left knee demonstrating severe medial compartment osteoarthritis. (B) Preoperative posterior-anterior view of the left knee demonstrating severe patellofemoral osteoarthritis. (A) Preoperative axial (skyline) view of the left knee demonstrating patellofemoral osteoarthritis with joint space narrowing, subchondral sclerosis, and osteophyte formation, with no acute fractures identified. (B) Preoperative posterior-anterior (Rosenberg) view of the left varus knee, demonstrating severe medial compartment osteoarthritis with joint space narrowing, subchondral sclerosis, and osteophyte formation, with no acute fractures identified.

A 68-year-old male with a BMI of 39.9, a past medical history of hypertension, and a prior right TKA performed by another surgeon, presented to the orthopedic clinic with complaints of left knee pain that worsened with walking and prolonged standing over the course of several months. Due to the patient's ongoing symptoms, radiograph films of the knee were obtained, which demonstrated left knee OA (Figures 1-2). Based on the Kellgren-Lawrence classification system, it was determined that this patient presented with stage III left knee OA with associated varus deformity. The patient reported that conservative measures had failed, and he expressed interest in definitive treatment with surgery. However, he did convey concerns due to a challenging postoperative course following his previous right TKA. At the time of the prior right TKA, the patient exhibited stage III right knee OA with varus alignment and comparable clinical symptoms. His previous right TKA was performed using a medial parapatellar approach under general anesthesia with a peripheral nerve block for regional anesthesia. The procedure was completed without intraoperative complications or technical difficulty, and estimated blood loss was minimal. The patient reported experiencing the worst pain he had ever felt on postoperative day (POD)-1, which he rated as a 10/10 on the visual analogue scale (VAS). The pain was not adequately controlled by the prescribed pain medications, including opioids. He required overnight hospital admission, and ambulation with a walker was achieved on POD-4 through at-home physical therapy. Additionally, the patient reported significant difficulty with physical therapy and experienced a notably prolonged recovery period before being able to ambulate without a walker due to the severe pain.

**Figure 3:**
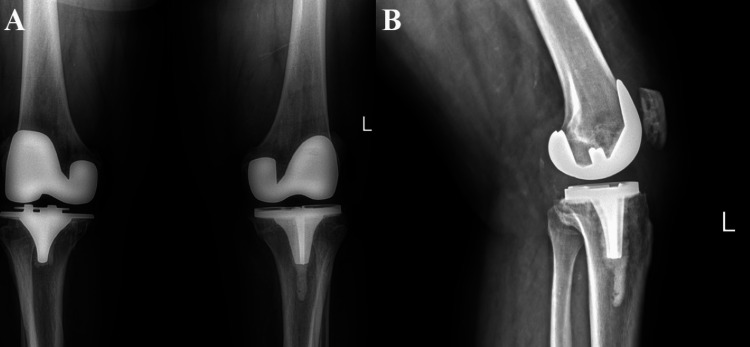
(A) POD-15 anteroposterior demonstrating well-positioned bilateral total knee arthroplasty. (B) POD-15 lateral radiograph demonstrating a well-positioned left total knee arthroplasty. (A) POD-15 anteroposterior X-ray demonstrates a well-positioned total knee arthroplasty bilaterally with stable osseous integration and no signs of loosening. (B) POD-15 lateral X-ray demonstrates a well-positioned total knee arthroplasty in the left knee with stable osseous integration and no signs of loosening. POD, postoperative day

The patient was objectively counseled on the risks and benefits of various surgical approaches, including the potential advantages of the medial subvastus technique for TKA. Following a shared decision-making discussion, the patient and surgeon decided to proceed with a left TKA using the medial subvastus approach. Given the surgeon's training and expertise with the technique and the patient being a good fit for the approach, it was selected to potentially minimize postoperative pain, promote early ambulation, and improve overall recovery. Subsequently, the patient underwent a left TKA with a medial subvastus approach performed under general anesthesia with an adjunctive peripheral nerve block for regional anesthesia. The operation proceeded without any intraoperative complications or technical challenges, and blood loss remained minimal throughout the case. The patient reported a significantly improved postoperative course compared to his previous right TKA. He rated his pain as a 3/10 on the VAS, describing it as manageable. The patient was able to walk on the same day and did not require overnight hospitalization. At follow-up, POD-15, the patient was able to ambulate up stairs, drive, walk down the hall without the use of a walker, and squat without pain. The patient only reported minimal muscle soreness and weakness. Follow-up X-rays demonstrated excellent implant placement in the left knee (Figure 3). The patient continued with physical therapy and reported only mild tightness behind the knee. He reported no adverse symptoms or effects. In a follow-up phone call with the patient on POD-70, he expressed high satisfaction with the results and the overall outcome of the second TKA, along with no adverse effects.

## Discussion

Definitive treatment for advanced knee OA is a TKA, which is traditionally performed with the medial parapatellar approach; however, alternative approaches such as the medial subvastus can be used [10,11]. The medial subvastus approach can offer benefits in the early postsurgical period, including reduced pain levels [11]. In this patient, his advanced knee OA, which can be seen in Figures 1-2, necessitated surgical intervention. After shared decision-making between the patient and surgeon, the medial subvastus approach to the TKA was selected due to the difficult postoperative period that he had experienced with his previous medial parapatellar approach TKA. Although the patient experienced a notably improved postoperative course following the medial subvastus approach, it is important to consider that other perioperative factors may have contributed to this outcome. Factors include inter-surgeon variability, surgical expertise, pain management strategies, physical therapy regimen, rehabilitation intensity, and potential psychological influences related to the first TKA. The contribution of the surgical approach alone cannot be definitively isolated as the cause of improved early postoperative outcomes in this case; however, it may play a crucial role.

Traditionally, the medial parapatellar approach has been regarded as the standard approach due to the ability to have excellent exposure of the knee joint, which allows surgeons to make precise bone cuts and appropriate prosthesis placement [12]. In the medial parapatellar approach, the vastus medialis obliquus (VMO) and extensor retinaculum are typically split, which can disrupt the extensor mechanism [13]. Additionally, the blood supply of the patella may be significantly disrupted, which can lead to increased anterior knee pain [14]. A medial parapatellar approach was used in the patient's first TKA, and he had a difficult recovery. The disruption of the extensor mechanisms and the patella blood supply may have played a role in the immense amount of pain, which delayed walking and created a difficult postoperative period. Additionally, confounding factors may have contributed to the increased postoperative difficulties following the medial parapallter approach TKA, including inadequate analgesia care, the possible need for a more extensive soft tissue release, or differences in postoperative rehabilitation protocols. The medial subvastus approach is an alternative TKA technique that preserves the knee extensor mechanism and minimizes vascular damage to the knee [11,12]. The medial subvastus approach preserves the integrity of the extensor mechanism by avoiding splitting of the VMO [11,15]. Instead, the VMO is retracted laterally, helping to maintain quadriceps strength and vascular integrity [11,15]. The medial subvastus approach is known for its ability to give earlier recovery due to less postoperative pain and early mobilization [11,15]. Given the potential benefits of a medial subvastus approach, after discussion with the patient, this approach was selected in an attempt to reduce postoperative pain levels.

Previous literature has demonstrated that the medial subvastus approach offers several notable advantages in the early postoperative period in comparison to the medial parapatellar approach [11]. Advantages include a shorter time to achieve active straight leg raise, higher functional scores, and improved early postoperative pain control [16]. Additionally, improved range of motion and a reduced need for lateral retinacular release have also been cited [12]. A study conducted by Berstock et al. demonstrated faster SLR recovery, lower visual analog scale (VAS) pain scores, improved total ROM at one week, fewer lateral releases, and less peri-operative blood loss [11]. However, Berstock et al. did find prolonged operative times with the medial subvastus approach [11]. Despite early postoperative benefits, previous literature indicates that long-term outcomes of the subvastus and medial parapatellar approaches are similar [11,12,16]. There were no significant long-term differences in pain or functional outcomes between the two approaches at extended follow-up [11,12,16]. Furthermore, Berstock et al. reported no difference in rates of adverse events, including infections, deep vein thrombosis, and postoperative stiffness requiring manipulation under anesthesia [11]. Given that the long-term outcomes between the two approaches are similar, the medial subvastus approach may offer particular value for patients who are at a greater risk for difficult postoperative recovery. The medial subvastus approach is a useful option when pain management, early mobility, and functional recovery are clinical priorities, allowing for a more individualized approach to patient care [11]. Both approaches are adequately equipped to use the same prostheses, including common TKA prosthesis designs, the cruciate-retaining (CR) model, and the posterior-stabilized (PS) design [8]. The CR model relies on a preserved posterior cruciate ligament for flexion stability [8]. The PS design features a femoral cam engaging a tibial polyethylene post to enhance stability during flexion [8].

In this case, the patient had a remarkable recovery with the medial subvastus TKA compared to his previous medial parapatellar TKA. Post-operative X-rays, Figure 3, demonstrate excellent implant placement. The patient reported improvement in pain, range of motion, quicker time to ambulation, prompt use of stairs, and an early ability to squat. It is well documented in existing literature that the subvastus approach has short-term post-operative benefits [11,12,16]. Although this case cannot definitively attribute improved outcomes solely to the surgical approach, the patient's lower self-reported VAS pain score and lack of need for overnight admission may be suggestive of short-term post-operative benefits, consistent with prior reports.

Creating definitive conclusions about the postoperative differences between the medial subvastus and medial parapatellar approaches based on a single patient is limited. In the future, randomized controlled trials involving patients with severe bilateral knee OA, where one knee undergoes TKA via the medial subvastus approach and the other via the medial parapellar approach, performed by the same surgeon trained in both techniques, would help minimize variability and better isolate the effect of the surgical approach. Accounting for potential confounding factors in such a study design would allow for more concrete comparisons and conclusions to be made.

## Conclusions

This case explores the potential benefits of the medial subvastus approach in TKA, particularly for patients with a history of challenging postoperative recovery following a traditional medial parapatellar approach. In this instance, the patient had undergone both a medial parapatellar TKA and a subvastus TKA. He experienced a significantly improved recovery course with reduced postoperative pain, earlier mobilization, and a quicker return to daily activities following the subvastus approach. Although a definitive statement cannot be made due to confounding variables in this patient, this patient's improvements are consistent with recent literature, suggesting the subvastus approach may offer superior early postoperative outcomes compared to the medial parapatellar approach. While both approaches offer comparable long-term results, the medial subvastus approach should be considered in patients to optimize early recovery and minimize postoperative complications. Orthopedic surgeons should consider this alternative approach, especially in patients with prior adverse experiences or concerns regarding early TKA recovery. Through evaluating patients’ prior surgical experiences and tailoring the TKA approach to the patient, patient outcomes and satisfaction may be enhanced.
